# Transfer learning in ECG diagnosis: Is it effective?

**DOI:** 10.1371/journal.pone.0316043

**Published:** 2025-05-19

**Authors:** Cuong V. Nguyen, Cuong D. Do

**Affiliations:** 1 College of Engineering and Computer Science, VinUniversity, Hanoi, Vietnam; 2 VinUni-Illinois Smart Health Center, VinUniversity, Hanoi, Vietnam; University of Essex, UNITED KINGDOM OF GREAT BRITAIN AND NORTHERN IRELAND

## Abstract

The adoption of deep learning in ECG diagnosis is often hindered by the scarcity of large, well-labeled datasets in real-world scenarios, leading to the use of transfer learning to leverage features learned from larger datasets. Yet the prevailing assumption that transfer learning consistently outperforms training from scratch has never been systematically validated. In this study, we conduct the first extensive empirical study on the effectiveness of transfer learning in multi-label ECG classification, by investigating comparing the fine-tuning performance with that of training from scratch, covering a variety of ECG datasets and deep neural networks. Firstly, We confirm that fine-tuning is the preferable choice for small downstream datasets; however, it does not necessarily improve performance. Secondly, the improvement from fine-tuning declines when the downstream dataset grows. With a sufficiently large dataset, training from scratch can achieve comparable performance, albeit requiring a longer training time to catch up. Thirdly, fine-tuning can accelerate convergence, resulting in faster training process and lower computing cost. Finally, we find that transfer learning exhibits better compatibility with convolutional neural networks than with recurrent neural networks, which are the two most prevalent architectures for time-series ECG applications. Our results underscore the importance of transfer learning in ECG diagnosis, yet depending on the amount of available data, researchers may opt not to use it, considering the non-negligible cost associated with pre-training.

## Introduction

Electrocardiogram (ECG) signals play a critical role in the early detection and diagnosis of cardiovascular diseases. The integration of automatic ECG interpretation, fueled by digitization and deep learning, has demonstrated performance on par with cardiologists [1, 2]. A major challenge to wide-scale adaptation of deep learning to ECG diagnosis is the lack of large-scale, high-quality labeled datasets in most real-world scenarios, due to prohibitive collection and annotation costs. To overcome this challenge, transfer learning is commonly employed, where features and parameters learned from a large dataset are reused and fine-tuned on a typically smaller, new dataset. This technique has been adapted from computer vision [3–7] to the ECG domain. Some studies have applied transfer learning to classify ECG arrhythmia by borrowing pre-trained weights on 2-D ImageNet [8], after transforming 1-D ECG signals to 2-D representations. For example, Salem *et al*. [9] and Tadesse *et al*. [10] generated 2-D spectrograms from ECG using Fourier Transform and applied pre-trained weights of DenseNet [11] and inception-v3 GoogLeNet [12] to diagnose cardiovascular diseases. Gajendran *et al*. [13] and Venton *et al*. [14] leveraged scalogram for 2-D conversion and fine-tuning convolutional neural networks (CNNs) [11, 12, 15–18] to classify ECG records. Zhang *et al*. [19] applied Hilbert Transform and Wigner-Ville distribution [20, 21] to convert signals to 2-D then using pre-trained ResNet101 [16] to build their classifiers.

Additionally, applying transfer learning directly to 1-D signals has shown encouraging results. Strodthoff *et al*. [22] reported significant improvements when pre-training *xresnet1d101* [23] on the PTB-XL [24] dataset and subsequently fine-tuning on smaller datasets. Weimann *et al*. [25] achieved up to a 6.57% improvement in the classification performance of Atrial Fibrillation using CNNs, pre-trained on the large Icentia11K dataset [26]. Jang *et al*. [27] showed that pre-training a convolutional autoencoder on the AUMC ICU dataset [28] of size 26,481 worked better than training from random initialization on the 10,646-sample dataset of the Shaoxing People’s Hospital of China [29]. Other studies have also reported positive results of transfer learning [30–35].

While previous 1-D approaches mentioned above have demonstrated the effectiveness of transfer learning in ECG diagnosis, these studies often focused on specific datasets and model architectures. For example, [25] reported an improvement of 6.57% with CNN pre-trained on the Icentia11K dataset, but several questions might be posed: Would the improvement have been significant with different deep learning models, such as LSTM [36] or GRU [37]? How did the target dataset size affect the improvement? Did fine-tuning in this case converge faster than training from scratch? There is a lack of systematic validation regarding the superiority of transfer learning over training from scratch. An implicit assumption is that transferring knowledge from a large upstream dataset consistently improves downstream performance on another dataset, compared to training from random initialization (scratch). However, this hypothesis has not been systematically verified. In this study, we aim to validate the hypothesis by testing it across different ECG datasets and deep learning architectures. Specifically, we conduct extensive experiments using three upstream datasets for pre-training models and five downstream datasets for fine-tuning pre-trained models. We employ six deep learning models, encompassing the two predominant architectures for ECG diagnosis: Convolutional Neural Networks [38–46] and Recurrent Neural Networks (RNNs) [42, 45, 47–52]. The comparison between fine-tuning performance and training from scratch provides insights into the effectiveness of transfer learning in ECG applications. Our key contributions and findings are as follows:

- We conduct the first extensive study on the effectiveness of transfer learning in the ECG domain, including six popular DNN architectures and five ECG datasets.
- Contrary to expectations, fine-tuning does not consistently outperform training from scratch. Its advantages diminish as the size of the downstream dataset increases.
- Fine-tuning can accelerate convergence, whereas training from scratch generally requires a longer time to sufficiently converge.
- For ECG data, fine-tuning demonstrates greater effectiveness with CNNs than with RNNs.

## Materials and methods

### Datasets

We used five publicly available ECG datasets in this work. The first was PTB-XL [53], containing 21,837 ECG records from 18,885 patients, covering 44 diagnostic statements. Signals were sampled at either 500 Hz or 1000 Hz, with a duration of ten seconds each. The 44 labels were categorized into five superclasses, namely: NORM (normal ECG), MI (Myocardial Infarction), STTC (ST/T-Changes), HYP (Hypertrophy), and CD (Conduction Disturbance) [22]. We focused on these five superclasses when conducting experiments with this dataset.

The second dataset was from the China Physiological Signal Challenge 2018 (CPSC2018) [54], including 6,877 ECG records, sampled at 500 Hz and lasted for 6–60 seconds each. There are nine diagnostic labels: NORM, AF (Atrial Fibrillation), I-AVB (First-degree atrioventricular block), LBBB (Left Bundle Branch Block), RBBB (Right Bundle Branch Block), PAC (Premature Atrial Contraction), PVC (Premature ventricular contraction), STD (ST-segment Depression), and STE (ST-segment Elevated).

The third was the Georgia dataset [55], consisting of 10,344 ECG signals with 10 seconds in length and a sampling rate of 500 Hz. The dataset has a diverse range of 67 unique diagnoses. However, our research concentrated on a subset of 10 specific labels having the highest number of samples: NORM, AF, I-AVB, PAC, SB (Sinus Bradycardia), LAD (left axis deviation), STach (Sinus Tachycardia), TAb (T-wave Abnormal), TInv (T-wave Inversion), and LQT (Prolonged QT interval).

The fourth was the PTB Diagnostic ECG Database [24], containing 549 ECG records sampled at 1000 Hz. We focused on two diagnostic classes: Myocardial Infarction (MI) and Healthy controls (NORM), covering 200 over 268 subjects involved in this dataset(it is worth noting that while there are ECG records from 290 subjects, clinical summaries are available for only 268 of them).

The last source was the Ribeiro dataset [1]. This contains 827 ECG records with seven annotations: NORM, I-AVB, RBBB, LBBB, SB, AF, and STach.

We reduced the sampling frequency of all ECG records to 100 Hz. This helps reduce computational load while retaining essential information. In addition, all records need to have the same duration. Since most ECG signals in the five datasets lasted for ten seconds, we used this as the desired duration. For records exceeding this timeframe, we applied cropping. For shorter records, since they only account for a tiny fraction, specifically six out of 6,877 records in the CPSC2018 dataset and 52 out of 10,334 records in the Georgia dataset, we simply omitted them. Each dataset was then split into training and test subsets with a test size ratio of 0.33. The split was done based on a patient-wise basis, records from the same patient were used exclusively in either the training or the test set, but not both. Table 1 summarizes the five datasets used in this work.

**Table 1:** Datasets used in this work.

Dataset	Labels used	Samples	Training samples	Testing samples
PTB-XL [53]	5	21,837	17,441	2,203
CPSC2018 [54]	9	6,877	4,603	2,268
Georgia [55]	10	10,344	6,895	3,397
PTB [24]	2	549	349	173
Ribeiro [1]	7	827	554	273

### Experiment settings

#### Evaluation metric.

We evaluated model performance on a dataset using their average *f*_1_ on the test subset across all labels, weighted by the number of samples belonging to each label in the test subset. Importantly, the test subset was only used for evaluation during each training epoch and was never employed to update the model’s parameters, ensuring prevention of patient-wise data leakage [56].

#### Pre-training.

In this work, we examined six DNN architectures. Three of these were convolutional: ResNet1d18, ResNet1d50, and ResNet1d101, which were adapted from the original 2-D versions [16]. The other three were recurrent DNNs: Long Short Term Memory (LSTM) [36], Bidirectional LSTM [36], and Gated Recurrent Unit (GRU) [37]. Three datasets were used for pre-training: PTB-XL, CPSC2018, and Georgia, due to their substantial sample sizes. Each of the six models was pre-trained on the training subset of each dataset for 100 epochs. We evaluated each model on the test subset during training, and only the checkpoint that achieved the best evaluation metric over 100 epochs was saved as the pre-trained model. We opted not to save the last checkpoint at the $100^{\text{th}}$ epoch due to observed overfitting as training progressed, especially for the three recurrent models. The other hyperparameters used are as follows: batch size 256, Adam optimizer [57] with learning rate 0.01, running average coefficients β=(0.9,0.999), hidden size 100 and dropout rate 0.3 for the three RNNs. Due to the computational cost involved, we were unable to explore how different set of hyperparameters might affect model performances. However, given the long training period of 100 epochs and the robustness of the Adam optimizer, it is reasonable to assume that our chosen hyperparameters is representative.

Training a model from scratch involved the same process described above and was applied to all five datasets, not limited to the three largest ones.

#### Fine-tuning.

**Fig 1:**
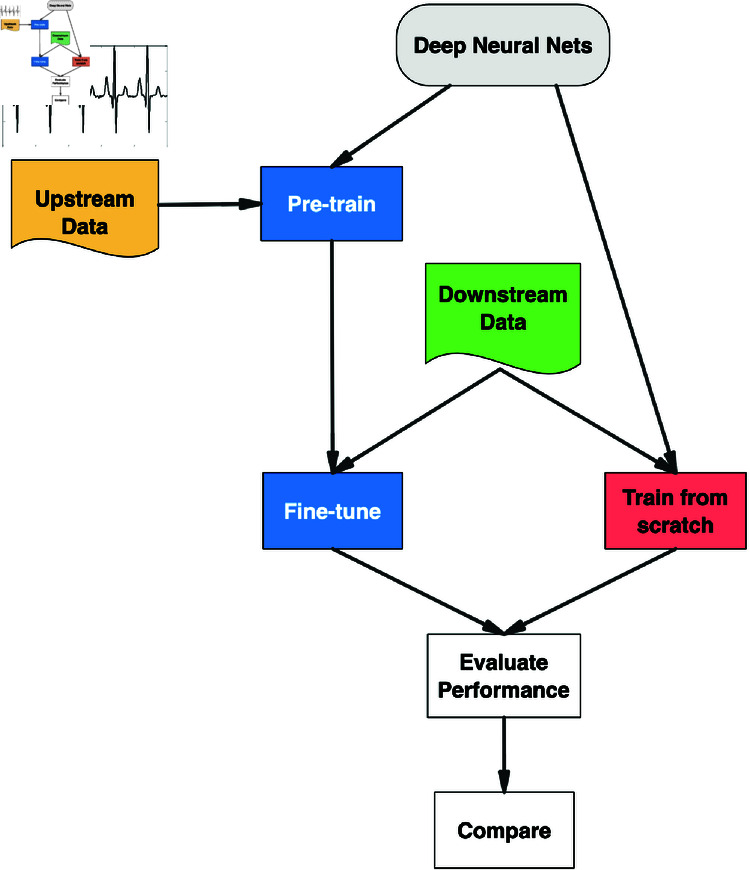
Experiment flowchart.

When fine-tuning a pre-trained model on a downstream dataset, as the number of output neurons may be different, we replaced the top fully-connected layer in the pre-trained model with a new layer with the number of neurons equal to the number of labels in the downstream dataset. For example, when fine-tuning ResNet1d18, which was pre-trained on PTB-XL (five labels), on Ribeiro as the downstream dataset (seven labels), we replaced the top layer with five outputs with a new one with seven outputs, and kept the layer’s input unchanged. Then the whole model underwent the same training procedure as pre-training, described in Sect Sec pretraining. Fig 1 visualizes the experiment flow.

## Results

### Fine-tuning does not necessarily improve performance

**Fig 2:**
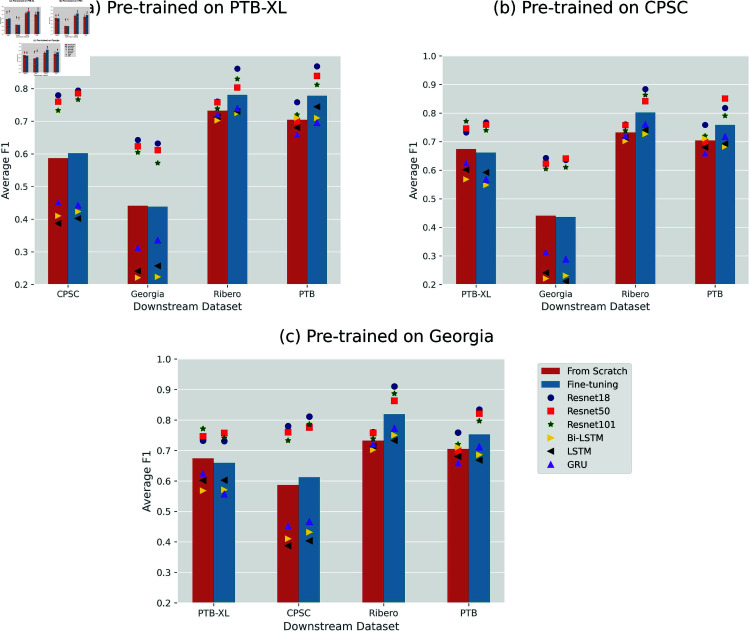
Performance comparison of fine-tuning and training from scratch, with three upstream datasets, six models, and four downstream datasets. In each chart, six symbols depict the average *f*_1_-scores for the respective models, and the bar shows the mean average score across these six models.

Fig 2 illustrates the performance comparison between fine-tuning and training from scratch. Each chart corresponds to one of the three upstream datasets, with the results of all six models on each downstream dataset scattered for both cases. The bars denote the average performance across the six models, providing the overall comparison.

Clearly, transfer learning does not consistently outperform training from scratch. On one hand, it significantly improved the model’s performance on Ribeiro and PTB, the two small downstream datasets. On the other hand, when using Georgia as the downstream dataset, there is little average difference in performance, despite variations among individual models. This is depicted in Fig 2a for PTB-XL and Fig 2b for CPSC2018 as upstream datasets. Notably, when fine-tuning on PTB-XL, the overall performance is slightly poorer than training from random initialization, regardless of whether pre-training occurred on CPSC2018 or Georgia, as seen in Figs 2b and 2c.

**Fig 3:**
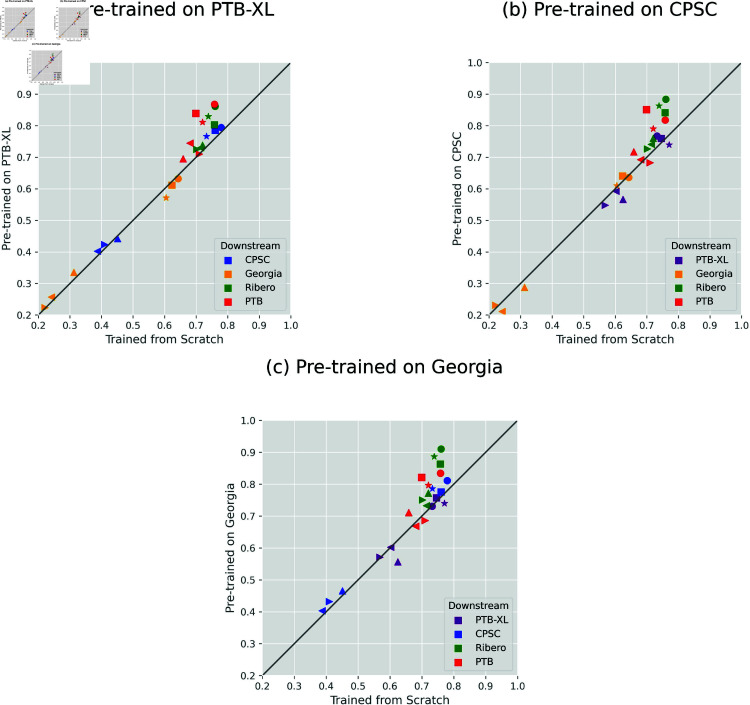
Another view of average-*f*_1_ comparison between fine-tuning (vertical axis) and training from scratch (horizontal axis). Each point corresponds to a specific model and downstream dataset combination. Model legend is the same as in Fig 2. Best viewed in color. That the majority of points lying above the identity line suggests that fine-tuning generally outperformed training from scratch. However, this is not always true.

Fig 3 shows an alternative perspective on the comparison, using the same model legend as in Fig 2. Each point on the plot represents a model and downstream dataset combination. In the scenario of pre-training on PTB-XL (Fig 3a), nearly all points remained above the identity line, indicating the superior performance of fine-tuning. However, after pre-training on CPSC2018 and Georgia datasets, fine-tuning RNNs mostly led to poorer results, as more triangular symbols (representing RNNs) fell below the line, especially for PTB-XL as the downstream dataset (shown as purple points). This observation aligns with the results shown in Fig 2.

### Fine-tuning improvement fades with downstream dataset size

The results presented in Sect Fine-tuning does not necessarily improve performance suggest that the comparison between fine-tuning and training from scratch is influenced by the size of the downstream dataset. Fine-tuning exhibited the most significant improvement over training from scratch when the dataset size was small (as observed in the cases of Ribeiro and PTB), with diminishing improvement as larger datasets (PTB-XL, CPSC2018, and Georgia) were used. To gain better insights, we conducted experiments with three pre-trained ResNets on the Georgia dataset. For the downstream task, we varied the size of the PTB-XL training set from 500 to 9000 samples, measuring the average *f*_1_ improvement achieved by fine-tuning over training from scratch. To ensure a fair comparison, evaluation was conducted on the same PTB-XL test subset used in Sect Fine-tuning does not necessarily improve performance, regardless of the number of training samples.

**Fig 4:**
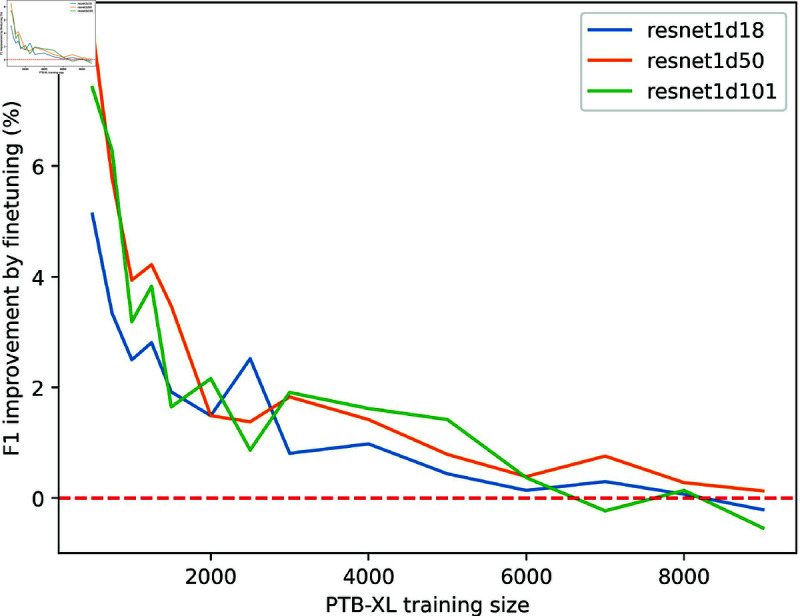
Fine-tuning improvement of the three ResNets with varying downstream dataset size.

Fig 4 shows that performance gain through fine-tuning declined as the training size increased. The most significant improvement occurred with a downstream dataset of 500 training samples, and training from scratch gradually reached comparable performance when the size reached 6000 samples. Though fluctuations were present in the region of fewer than 2000 samples, likely because of the inherent randomness in deep learning [58], overall the declining trend remains evident. A reasonable explanation for the trend is that for small target datasets, training from scratch is more prone to overfitting, leading to poor generalization; thus latent features learned from pre-training would likely enhance the model’s robustness. In contrast, when the target dataset is large enough, overfitting is alleviated, and pre-trained latent features tend to be “overwritten” by new features, which quickly diminishes the fine-tuning benefits. To conclude, the results highlight the importance of transfer learning in the small dataset regime, though it may be less necessary when dealing with sufficiently large datasets.

### Fine-tuning can accelerate convergence

**Fig 5:**
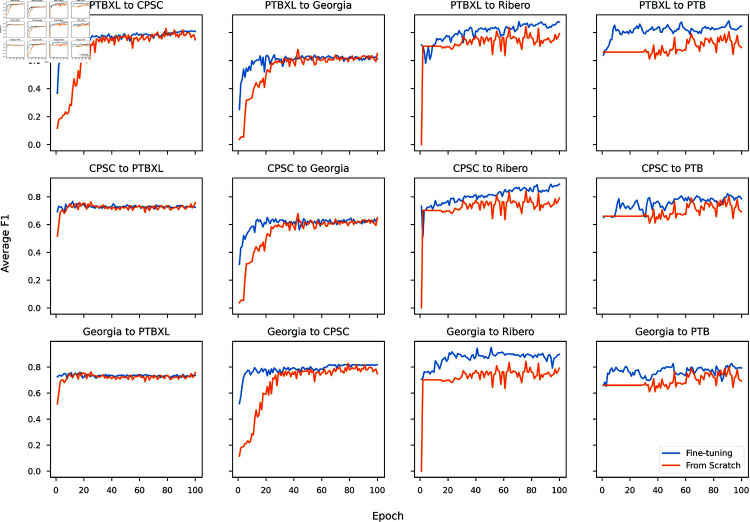
Performances of ResNet1d18 during fine-tuning and training from scratch. Three rows represent three upstream datasets: PTB-XL, CPSC2018, and Georgia, respectively.

While transfer learning might not consistently outperform training from scratch in terms of accuracy, the next question is whether it contributed to speeding up the training process. We examined the evaluation metric across 100 epochs in all cases to answer this question. Fig 5 shows ResNet1d18’s average *f*_1_ at each training epoch on the corresponding downstream test subset. Notably, in scenarios such as transferring from PTB-XL to CPSC2018, from PTB-XL to Georgia, from CPSC2018 to Georgia, and from Georgia to CPSC2018, although the performance of training from scratch eventually caught up with that of fine-tuning, it took approximately 30-35 epochs to do so. Meanwhile, transferring from CPSC2018 to PTB-XL or from Georgia to PTB-XL offered minor accelerating benefits, and for small downstream datasets (Ribeiro, PTB), transfer learning was clearly superior. The accelerated pattern of fine-tuning is primarily due to the latent ECG representations learned during the pre-training process. When training from scratch, model parameters are initialized randomly, requiring the optimizer to spend more time exploring the parameter space. Conversely, fine-tuning benefits from the optimal parameters obtained through pre-training, providing a head start and enabling faster convergence. This implies that fine-tuning could be beneficial in applications where faster training is preferred or required, such as continual learning [59]. Additionally, much more fluctuated convergences were observed when the target data were Ribeiro and PTB. It is potentially because of the small size of testing data (273 and 173 samples, respectively, compared to 2000-3000 testing samples of other datasets), which may have led to higher variation due to randomness. For results of other models, please refer to S1 Appendix.

### Fine-tuning tends to work better with CNNs than with RNNs

Concerning architectural selection, not only achieving higher overall *f*_1_ than LTSM, Bi-LSTM, and GRU, three ResNet models (the circle, the square, and the star in Fig 2) showed better compatibility with transfer learning. Fine-tuning those CNNs consistently resulted in improved performance compared to training from scratch in almost all scenarios, no matter which upstream and downstream datasets were used, as shown in Figs 2 and 3. In contrast, transfer learning had a minor impact on the three RNNs, as in numerous cases, their performance even lagged behind that of random initialization (see the up, left, and right triangles in the two figures). Moreover, when examining the convergence patterns (refer to Fig 5 for ResNet18 and five figures in S1 Appendix for other models), it is clear that fine-tuning CNNs played a crucial role in expediting and stabilizing the convergence process, whereas RNNs (especially GRU) exhibited a notably more erratic result.

This phenomenon can be explained by the inherent characteristics of the two architectures. Convolutional layers within CNNs are adept at capturing spatial features such as shapes, patterns, peaks, and troughs—features that are low-level and do not necessitate relearning during fine-tuning on downstream datasets On the other hand, LTSM and GRU specialize in capturing temporal dependencies, processing signals sequentially to maintain a “memory” that is high-level and complex. Consequently, the learned memory from one dataset may not be applicable or effective for others, rendering the transfer of such memory ineffective. Furthermore, inherent challenges in training RNNs, such as vanishing gradients [60] and exploding gradients [61], may exacerbate the difficulty of fine-tuning these networks.

## Conclusion

In this work, we empirically investigate the effectiveness of transfer learning in multi-label ECG diagnosis through extensive experiments involving diverse datasets and deep learning models. We show that when the downstream dataset is sufficiently large, pre-training may not exhibit superior performance compared to training from random initialization. This observation challenges the prevailing assumption that transfer learning invariably enhances performance across different tasks. Nevertheless, in many real-world scenarios, the availability of small downstream datasets is a common constraint due to the substantial costs associated with data collection and annotation. In such cases, we assert that transfer learning remains a crucial and valuable approach. Even when a decently large dataset is available, transfer learning will still be useful, as it can accelerate convergence, saving resources & time and expediting both research and production cycles.

Moreover, our results confirm that fine-tuning tends to yield more effective results with CNNs than with RNNs in ECG classification. Contrary to 2-D images, RNNs are also a potential method to process time-series ECG signals. However, as mentioned in Sect Fine-tuning tends to work better with CNNs than with RNNs, inherent designs of RNNs make it more difficult to transfer knowledge learned from one dataset to another. Even in the case of training from scratch, LSTM, Bi-LSTM, and GRU showed inferior performance than that of ResNets (Sect Fine-tuning does not necessarily improve performance). Thus we argue that in general, CNNs should be the preferred choice when deciding on architectures for ECG applications.

While CNNs still remain dominant, transformer, attention-based architectures have shown promising results in ECG diagnosis [62–64]. Future research could benefit from exploring how transfer learning enhances these networks. Additionally, as described in Sect Experiment settings our work considers the average *f*_1_-score as the evaluation metric, which provides a general view of the model’s performance across all cardiac categories. However, this metric limits the ability to gain in-depth and specific insights into individual labels. We aim to address this limitation in future work.

## Supporting information

S1 Appendix Full results Five figures for Sect Fine-tuning can accelerate and four tables for Sects Fine-tuning does not necessarily improve performance and Fine-tuning tends to work better with CNNs than with RNNs.

## References

[1] Ribeiro AH, Ribeiro MH, Paixão GMM, Oliveira DM, Gomes PR, Canazart JA, et al. Automatic diagnosis of the 12-lead ECG using a deep neural network. Nat Commun. 2020;11(1):1760. doi: 10.1038/s41467-020-15432-4 32273514 PMC7145824

[2] Hannun AY, Rajpurkar P, Haghpanahi M, Tison GH, Bourn C, Turakhia MP, et al. Cardiologist-level arrhythmia detection and classification in ambulatory electrocardiograms using a deep neural network. Nat Med. 2019;25(1):65–9. doi: 10.1038/s41591-018-0268-3 30617320 PMC6784839

[3] Yosinski J, Clune J, Bengio Y, Lipson H. How transferable are features in deep neural networks? Adv Neural Informat Proc Syst. 2014;27.

[4] Kornblith S, Shlens J, Le Q. Do better ImageNet models transfer better? In: Proceedings of the IEEE/CVF conference on computer vision and pattern recognition. 2019. p. 2661–71.

[5] Pan S, Yang Q. A survey on transfer learning. IEEE Trans Knowl Data Eng. 2009;22(10):1345–59.

[6] Donahue J, Jia Y, Vinyals O, Hoffman J, Zhang N, Tzeng E. Decaf: A deep convolutional activation feature for generic visual recognition. In: Proceedings of the international conference on machine learning. PMLR; 2014. p. 647–55.

[7] Sharif-Razavian A, Azizpour H, Sullivan J, Carlsson S. CNN features off-the-shelf: an astounding baseline for recognition. In: Proceedings of the IEEE conference on computer vision and pattern recognition workshops. 2014. p. 806–13.

[8] Deng J, Dong W, Socher R, Li L, Li K, Fei-Fei L. ImageNet: A large-scale hierarchical image database. In: 2009 IEEE conference on computer vision and pattern recognition. 2009. p. 248–55.

[9] Salem M, Taheri S, Yuan JS. ECG arrhythmia classification using transfer learning from 2-dimensional deep CNN features. In: 2018 IEEE biomedical circuits and systems conference (BioCAS). 2018. p. 1–4.

[10] Tadesse GA, Zhu T, Liu Y, Zhou Y, Chen J, Tian M, et al. Cardiovascular disease diagnosis using cross-domain transfer learning. In: 2019 41st Annual international conference of the IEEE engineering in medicine and biology society (EMBC). IEEE; 2019. p. 4262–5. 10.1109/EMBC.2019.8857737 31946810

[11] Huang G, Liu Z, Weinberger KQ. Densely connected convolutional networks. CoRR; 2016.

[12] Szegedy C, Liu W, Jia Y, Sermanet P, Reed S, Anguelov D, et al. Going deeper with convolutions; 2014.

[13] Gajendran M, Khan M, Khattak M. ECG classification using deep transfer learning. In: Proceedings of the 2021 4th international conference on information and computer technologies (ICICT). IEEE; 2021. p. 1–5.

[14] Venton J, Aston PJ, Smith NAS, Harris PM. Signal to image to classification: transfer learning for ECG. In: 2020 11th conference of the European study group on cardiovascular oscillations (ESGCO). IEEE; 2020. p. 1–2.

[15] Simonyan K, Zisserman A. Very deep convolutional networks for large-scale image recognition; 2015.

[16] He K, Zhang X, Ren S, Sun J. Deep residual learning for image recognition. In: Proceedings of the IEEE conference on computer vision and pattern recognition. 2016. p. 770–8.

[17] Redmon J. Darknet: Open source neural networks in C; 2013–2016.

[18] Tan M, Le Q. Efficientnet: Rethinking model scaling for convolutional neural networks. In: Proceedings of the international conference on machine learning. PMLR; 2019. p. 6105–14.

[19] Zhang Y, Li J, Wei S, Zhou F, Li D. Heartbeats classification using hybrid time-frequency analysis and transfer learning based on ResNet. IEEE J Biomed Health Inform. 2021;25(11):4175–84. doi: 10.1109/JBHI.2021.3085318 34077377

[20] Sultan Qurraie S, Ghorbani Afkhami R. ECG arrhythmia classification using time frequency distribution techniques. Biomed Eng Lett. 2017;7(4):325–32. doi: 10.1007/s13534-017-0043-2 30603183 PMC6208516

[21] Dhok S, Pimpalkhute V, Chandurkar A, Bhurane AA, Sharma M, Acharya UR. Automated phase classification in cyclic alternating patterns in sleep stages using Wigner-Ville Distribution based features. Comput Biol Med. 2020;119:103691. doi: 10.1016/j.compbiomed.2020.103691 32339125

[22] Strodthoff N, Wagner P, Schaeffter T, Samek W. Deep Learning for ECG Analysis: Benchmarks and Insights from PTB-XL. IEEE J Biomed Health Inform. 2021;25(5):1519–28. doi: 10.1109/JBHI.2020.3022989 32903191

[23] He T, Zhang Z, Zhang H, Zhang Z, Xie J, Li M. Bag of tricks for image classification with convolutional neural networks. In: Proceedings of the IEEE/CVF conference on computer vision and pattern recognition. 2019. p. 558–67.

[24] Bousseljot R, Kreiseler D, Schnabel A. Nutzung der EKG-Signaldatenbank CARDIODAT der PTB über das Internet; 1995.

[25] Weimann K, Conrad TOF. Transfer learning for ECG classification. Sci Rep. 2021;11(1):5251. doi: 10.1038/s41598-021-84374-8 33664343 PMC7933237

[26] Tan S, Androz G, Chamseddine A, Fecteau P, Courville A, Bengio Y. Icentia11k: An unsupervised representation learning dataset for arrhythmia subtype discovery. arXiv Preprint. 2019. doi: 10.48550/arXiv.1910.09570

[27] Jang J-H, Kim TY, Yoon D. Effectiveness of transfer learning for deep learning-based electrocardiogram analysis. Healthc Inform Res. 2021;27(1):19–28. doi: 10.4258/hir.2021.27.1.19 33611873 PMC7921576

[28] Lee S, Park J, Kim D, Kim T, Park R, Yoon D. Constructing a bio-signal repository from an intensive care unit for effective big-data analysis. In: Proceedings of the 14th ACM conference on embedded network sensor systems CD-ROM. 2016. p. 372–3.

[29] Zheng J, Zhang J, Danioko S, Yao H, Guo H, Rakovski C. A 12-lead electrocardiogram database for arrhythmia research covering more than 10,000 patients. Sci Data. 2020;7(1):48. doi: 10.1038/s41597-020-0386-x 32051412 PMC7016169

[30] Chen L, Xu G, Zhang S, Kuang J, Hao L. Transfer learning for electrocardiogram classification under small dataset. In: Machine learning and medical engineering for cardiovascular health and intravascular imaging and computer assisted stenting: First international workshop, MLMECH 2019, and 8th Joint international workshop, CVII-STENT 2019, Held in Conjunction with MICCAI 2019. 2019. p. 45–54.

[31] Ghaffari A, Madani N. Atrial fibrillation identification based on a deep transfer learning approach. Biomed Phys Eng Express. 2019;5(3):035015. doi: 10.1088/2057-1976/ab1104

[32] Li K, Du N, Zhang A. Detecting ECG abnormalities via transductive transfer learning. In: Proceedings of the ACM conference on bioinformatics, computational biology and biomedicine. 2012. p. 210–7.

[33] Nguyen CV, Duong HM, Do CD. MELEP: A novel predictive measure of transferability in multi-label ECG diagnosis. J Healthc Inform Res. 2024;1–17. 39131101 10.1007/s41666-024-00168-3 PMC11310184

[34] Jin BT, Palleti R, Shi S, Ng AY, Quinn JV, Rajpurkar P, et al. Transfer learning enables prediction of myocardial injury from continuous single-lead electrocardiography. J Am Med Inform Assoc. 2022;29(11):1908–18. doi: 10.1093/jamia/ocac135 35994003 PMC9552286

[35] Kumar LVR, Sai YP. A new transfer learning approach to detect cardiac arrhythmia from ECG signals. SIViP. 2022;16(7):1945–53. doi: 10.1007/s11760-022-02155-w

[36] Hochreiter S, Schmidhuber J. Long short-term memory. Neural Comput. 1997;9(8):1735–80. doi: 10.1162/neco.1997.9.8.1735 9377276

[37] Cho K, Van Merriënboer B, Gulcehre C, Bahdanau D, Bougares F, Schwenk H. Learning phrase representations using RNN encoder-decoder for statistical machine translation. arXiv Preprint. 2014.

[38] Cai C, Imai T, Hasumi E, Fujiu K. One-shot screening: utilization of a two-dimensional convolutional neural network for automatic detection of left ventricular hypertrophy using electrocardiograms. Computer Methods and Programs in Biomedicine. 2024:108097. 38428250 10.1016/j.cmpb.2024.108097

[39] Fan X, Yao Q, Cai Y, Miao F, Sun F, Li Y. Multiscaled fusion of deep convolutional neural networks for screening atrial fibrillation from single lead short ECG recordings. IEEE J Biomed Health Inform. 2018;22(6):1744–53. doi: 10.1109/JBHI.2018.2858789 30106699

[40] Li Y, Pang Y, Wang J, Li X. Patient-specific ECG classification by deeper CNN from generic to dedicated. Neurocomputing. 2018;314:336–46. doi: 10.1016/j.neucom.2018.06.068

[41] Wang J. A deep learning approach for atrial fibrillation signals classification based on convolutional and modified Elman neural network. Future Gener Comput Syst. 2020;102:670–9. doi: 10.1016/j.future.2019.09.012

[42] Petmezas G, Haris K, Stefanopoulos L, Kilintzis V, Tzavelis A, Rogers JA, et al. Automated atrial fibrillation detection using a hybrid CNN-LSTM network on imbalanced ECG datasets. Biomed Signal Process Control. 2021;63:102194. doi: 10.1016/j.bspc.2020.102194

[43] Baloglu UB, Talo M, Yildirim O, Tan RS, Acharya UR. Classification of myocardial infarction with multi-lead ECG signals and deep CNN. Pattern Recognit Lett. 2019;122:23–30. doi: 10.1016/j.patrec.2019.02.016

[44] Guo J, Li W, Huang H. An ECG detection device based on convolutional neural network. In: 2023 8th international conference on intelligent computing and signal processing (ICSP). 2023. p. 860–4.

[45] Limam M, Precioso F. Atrial fibrillation detection and ECG classification based on convolutional recurrent neural network. In: 2017 Computing in cardiology (CinC). IEEE; 2017. p. 1–4.

[46] Loh HW, Ooi CP, Oh SL, Barua PD, Tan YR, Molinari F, et al. Deep neural network technique for automated detection of ADHD and CD using ECG signal. Comput Methods Programs Biomed. 2023;241:107775. doi: 10.1016/j.cmpb.2023.107775 37651817

[47] Singh S, Pandey SK, Pawar U, Janghel RR. Classification of ECG arrhythmia using recurrent neural networks. Procedia Comput Sci. 2018;132:1290–7.

[48] Prabhakararao E, Dandapat S. Attentive RNN-based network to fuse 12-lead ECG and clinical features for improved myocardial infarction diagnosis. IEEE Signal Process Lett. 2020;27:2029–33.

[49] Kumar D, Peimankar A, Sharma K, Domínguez H, Puthusserypady S, Bardram JE. Deepaware: A hybrid deep learning and context-aware heuristics-based model for atrial fibrillation detection. Comput Methods Programs Biomed. 2022;221:106899. doi: 10.1016/j.cmpb.2022.106899 35640394

[50] Saadatnejad S, Oveisi M, Hashemi M. LSTM-based ECG classification for continuous monitoring on personal wearable devices. IEEE J Biomed Health Inform. 2020;24(2):515–23. doi: 10.1109/JBHI.2019.2911367 30990452

[51] Gutiérrez-Fernández-Calvillo M, Cámara-Vázquez M, Hernández-Romero I, Guillem M, Climent A, Fambuena-Santos C. Non-invasive estimation of Atrial Fibrillation driver position using long-short term memory neural networks and body surface potentials. Comput Methods Programs Biomed. 2024:108052. 38350188 10.1016/j.cmpb.2024.108052

[52] Faust O, Shenfield A, Kareem M, San TR, Fujita H, Acharya UR. Automated detection of atrial fibrillation using long short-term memory network with RR interval signals. Comput Biol Med. 2018;102:327–35. doi: 10.1016/j.compbiomed.2018.07.001 30031535

[53] Wagner P, Strodthoff N, Bousseljot RD, Kreiseler D, Lunze FI, Samek W, et al. PTB-XL, a large publicly available electrocardiography dataset. Sci Data. 2020;7(1):154. 32451379 10.1038/s41597-020-0495-6 PMC7248071

[54] Liu F, Liu C, Zhao L, Zhang X, Wu X, Xu X, et al. An open access database for evaluating the algorithms of electrocardiogram rhythm and morphology abnormality detection. J Med Imaging Health Inform. 2018;8(7):1368–73. doi: 10.1166/jmihi.2018.2442

[55] Perez Alday EA, Gu A, J Shah A, Robichaux C, Ian Wong A-K, Liu C, et al. Classification of 12-lead ECGs: the PhysioNet/Computing in cardiology challenge 2020. Physiol Meas. 2021;41(12):124003. doi: 10.1088/1361-6579/abc960 33176294 PMC8015789

[56] Kaufman S, Rosset S, Perlich C, Stitelman O. Leakage in data mining. ACM Trans Knowl Discov Data. 2012;6(4):1–21. doi: 10.1145/2382577.2382579

[57] Kingma DP, Ba J. Adam: A method for stochastic optimization. arXiv Preprint. 2014. doi: arXiv:1412.6980

[58] Doe J. Understanding the universe. J Astrophys. 2023;12(3):45–67. doi: 10.1234/astro.2023.001

[59] Kiyasseh D, Zhu T, Clifton D. A clinical deep learning framework for continually learning from cardiac signals across diseases, time, modalities, and institutions. Nat Commun. 2021;12(1):4221. doi: 10.1038/s41467-021-24483-0 34244504 PMC8270996

[60] Hochreiter S, Bengio Y, Frasconi P, Schmidhuber J. Gradient flow in recurrent nets: the difficulty of learning long-term dependencies; 2001.

[61] Bengio Y, Simard P, Frasconi P. Learning long-term dependencies with gradient descent is difficult. IEEE Trans Neural Netw. 1994;5(2):157–66. doi: 10.1109/72.279181 18267787

[62] Hammad M, Pl-awiak P, Wang K, Acharya U. ResNet-Attention model for human authentication using ECG signals. Expert Syst. 2021;38(6):e12547.

[63] Wang J, Qiao X, Liu C, Wang X, Liu Y, Yao L, et al. Automated ECG classification using a non-local convolutional block attention module. Comput Methods Programs Biomed. 2021;203:106006. doi: 10.1016/j.cmpb.2021.106006 33735660

[64] Natarajan A, Chang Y, Mariani S, Rahman A, Boverman G, Vij S. A wide and deep transformer neural network for 12-lead ECG classification. In: 2020 Computing in cardiology. IEEE; 2020. p. 1–4.

