# Supplementary material for: Transfer learning in ECG diagnosis: Is it effective?
Source: PLoS One. 2025 May 19;20(5):e0316043. doi: 10.1371/journal.pone.0316043 (PMC12088039; doi:10.1371/journal.pone.0316043)
Supplement: S1 Appendix Full results — Five figures for Sect Fine-tuning can accelerate and four tables for Sects Fine-tuning does not necessarily improve performance and Fine-tuning tends to work better with CNNs than with RNNs. [file pone.0316043.s001.pdf]

S1 Appendix

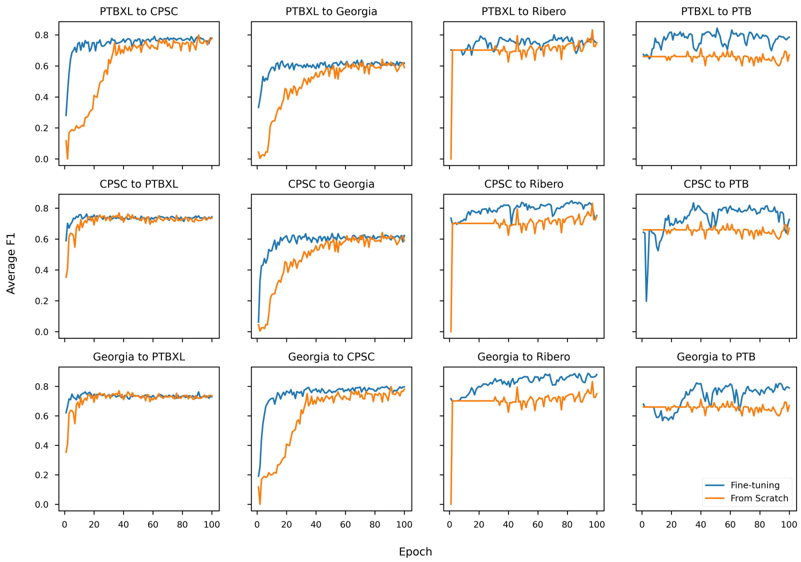

Fig 1. Performances of ResNet1d50 during fine-tuning and training from scratch.

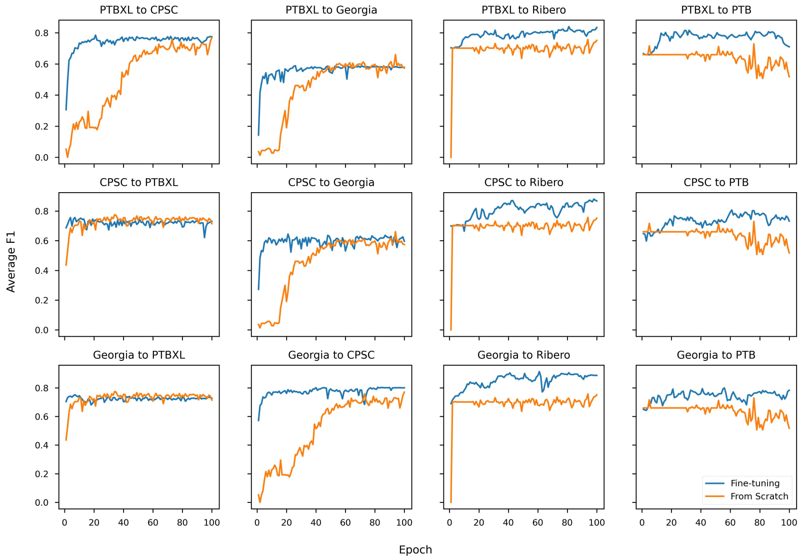

Fig 2. Performances of ResNet1d101 during fine-tuning and training from scratch.

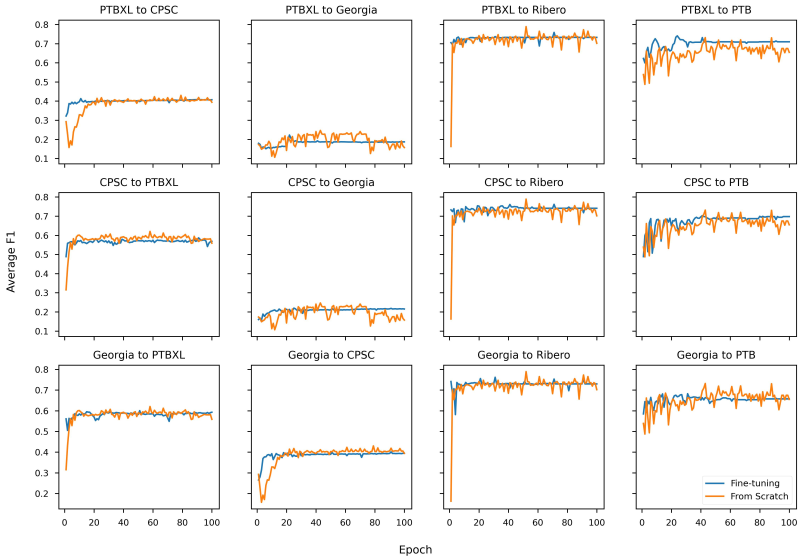

Fig 3. Performances of LSTM during fine-tuning and training from scratch.

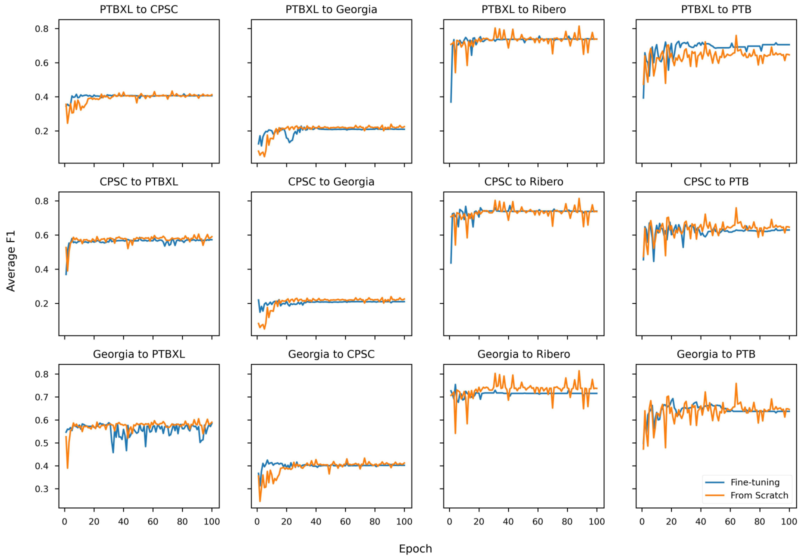

Fig 4. Performances of Bi-LSTM during fine-tuning and training from scratch.

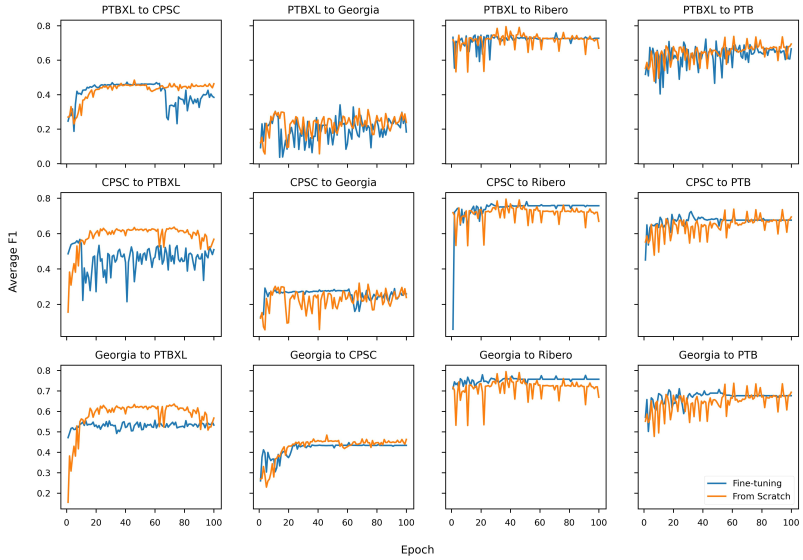

Fig 5. Performances of GRU during fine-tuning and training from scratch.

Table 1. Performance of training from scratch: maximum average f_1-score during 100 epochs.

	PTB-XL	CPSC	Georgia	Ribero	PTB
Resnet18	0.732	0.780	0.643	0.760	0.758
Resnet50	0.746	0.760	0.623	0.758	0.699
Resnet101	0.771	0.733	0.605	0.738	0.720
Bi-LSTM	0.568	0.410	0.221	0.702	0.710
LSTM	0.602	0.387	0.241	0.714	0.680
GRU	0.625	0.451	0.312	0.720	0.659

Table 2. Fine-tuning performance of models pre-trained on PTB-XL: maximum average f_1-score during 100 epochs.

	CPSC	Georgia	Ribero	PTB
Resnet18	0.794	0.632	0.861	0.868
Resnet50	0.786	0.612	0.803	0.839
Resnet101	0.766	0.572	0.829	0.811
Bi-LSTM	0.423	0.223	0.725	0.710
LSTM	0.402	0.257	0.730	0.745
GRU	0.442	0.335	0.739	0.695

Table 3. Fine-tuning performance of models pre-trained on CPSC: maximum average f_1-score during 100 epochs.

	PTB-XL	Georgia	Ribero	PTB
Resnet18	0.767	0.636	0.884	0.818
Resnet50	0.760	0.641	0.841	0.851
Resnet101	0.739	0.610	0.863	0.790
Bi-LSTM	0.548	0.231	0.726	0.683
LSTM	0.592	0.211	0.741	0.692
GRU	0.567	0.288	0.760	0.717

Table 4. Fine-tuning performance of models pre-trained on Georgia: maximum average f_1-score during 100 epochs.

	PTB-XL	CPSC	Ribero	PTB
Resnet18	0.731	0.811	0.910	0.834
Resnet50	0.757	0.775	0.863	0.821
Resnet101	0.740	0.786	0.887	0.797
Bi-LSTM	0.571	0.432	0.751	0.686
LSTM	0.602	0.403	0.733	0.668
GRU	0.557	0.466	0.772	0.711
